# Uncovering key steps in FGF12 cellular release reveals a common mechanism for unconventional FGF protein secretion

**DOI:** 10.1007/s00018-024-05396-9

**Published:** 2024-08-19

**Authors:** Martyna Biadun, Martyna Sochacka, Marta Kalka, Aleksandra Chorazewska, Radoslaw Karelus, Daniel Krowarsch, Lukasz Opalinski, Malgorzata Zakrzewska

**Affiliations:** 1 Department of Protein Engineering, Faculty of Biotechnology, University of Wroclaw, Joliot-Curie 14a, Wroclaw, 50-383 Poland; 2 Department of Protein Biotechnology, Faculty of Biotechnology, University of Wroclaw, Joliot-Curie 14a, Wroclaw, 50-383 Poland

**Keywords:** ATP1A1, FGF12, FGFs, FHFs, Unconventional protein secretion (UPS)

## Abstract

FGF12 belongs to a subfamily of FGF proteins called FGF homologous factors (FHFs), which until recently were thought to be non-signaling intracellular proteins. Our recent studies have shown that although they lack a conventional signal peptide for secretion, they can reach the extracellular space, especially under stress conditions. Here, we unraveled that the long “a” isoform of FGF12 is secreted in a pathway involving the A1 subunit of Na(+)/K(+) ATPase (ATP1A1), Tec kinase and lipids such as phosphatidylinositol and phosphatidylserine. Further, we showed that the short “b” isoform of FGF12, which binds ATP1A1 and phosphatidylserine less efficiently, is not secreted from cells. We also indicated regions in the FGF12a protein sequence that are crucial for its secretion, including N-terminal fragment and specific residues, and proposed that liquid-liquid phase separation may be important in this process. Our results strongly suggest that the mechanism of this process is very similar for all unconventionally secreted FGF proteins.

**Supplementary Information:**

The online version contains supplementary material available at 10.1007/s00018-024-05396-9.

## Introduction

FGF12 is the best-characterized representative of a subfamily of fibroblast growth factors (FGFs) called FGF homologous factors (FHFs), consisting of four proteins (FGF11, FGF12, FGF13 and FGF14), which until recently were considered exclusively intracrine proteins [1, 2]. FGF12 is mainly expressed in the developing and mature nervous system and other excitable tissues such as cardiac and longitudinal muscle myocytes, olfactory epithelium, but also in non-excitable cells such as fibroblasts [3–6]. Like the other FHF proteins, FGF12 undergoes alternative splicing, resulting in two isoforms. The longer isoform has a nuclear localization sequence (NLS) and localizes to the nucleus, while the shorter isoform is mainly observed in the cytoplasm [1]. FGF12 modulates voltage-gated ion channels, is involved in ribosomal biogenesis and regulates intracellular signaling [7–10]. We recently showed that FGF12 and all other FHF proteins are also capable of forming signaling hubs with FGF receptors on the cell surface [11, 12].

Most paracrine and all endocrine FGFs have a classical signal peptide (SP) for secretion that directs them to the conventional ER/Golgi-dependent secretory pathway. FGF1, FGF2 and FGF11-14 lack SP, but are nevertheless secreted through an unconventional mechanism [12–14]. There are four pathways of unconventional protein secretion (UPS), type I, II, III, and IV. UPS type I involves direct translocation across the plasma membrane and is utilized by proteins such as HIV-Tat [15, 16], Tau [17, 18] and FGF2 [13, 19]. UPS type II is dependent on the activity of ATP-binding cassette (ABC) transporters and is indicated in the export of some heat shock proteins (HSPs), such as HSP70 [20]. UPS type III employs intracellular vesicle intermediates such as autophagosomes or exosomes [13, 21]. Transmembrane proteins that reach the plasma membrane upon disruption of ER-Golgi transport through the Golgi bypass, employ UPS type IV [13].

In our recent study, we discovered that FHFs are secreted by an unconventional mechanism, as they are secreted even after the addition of brefeldin, an inhibitor of the ER/Golgi secretion pathway. We also found that their secretion was inhibited by ouabain, indicating a role of Na(+)/K(+) ATPase [12]. Here, we focused on identifying key components of FGF12’s secretion pathway and comparing them with the secretion mechanisms of other FGF proteins secreted in unconventional way.

## Materials and methods

### Antibodies and reagents

Anti-GFP (#50430-2-AP) antibody was obtained from Proteintech (Chicago, IL, USA). Anti-HSP70 (#4872) and anti-Tec (#4987) antibodies were purchased from Cell Signaling Technology (Danvers, MA, USA). Anti-ATP1A1 (ab-7671) antibody was from Abcam (Cambridge, UK). Anti-ATP1B1 (#HPA012911) antibody was obtained from Sigma-Aldrich (ST Louis, MO, USA). Anti-SBP (#sc-101595) antibody was from Santa Cruz Biotechnology (Dallas, TX, USA). Anti-His Tag (HIS.H8) (#WH329003) was obtained from Thermo Fisher Scientific (Waltham, MA, USA). Horseradish peroxidase-conjugated secondary antibodies (#115-035-003, #111-035-144) were from Jackson ImmunoResearch (Cambridge, UK). Pierce anti-c-myc-tag magnetic beads (#88842) and Pierce streptavidin magnetic beads (#88816) were obtained from Thermo Fisher Scientific. Ouabain octahydrate (#O125), brefeldin A (#B7651) and LFM-A13 (#435300) were from Sigma-Aldrich. Cyclosporin A (#FC20749) was purchased from Biosynth (Compton, UK). EDTA and DTT were from BioShop (Ontario, Canada). Paraformaldehyde (PFA) was purchased from Carl Roth (Karlsruhe, Germany). Buffer components were from Sigma-Aldrich.

### Cells

The human osteosarcoma (U2OS) cell line (#HTB-96) was purchased from American Type Culture Collection (ATCC, Manassas, VA, USA) and cultured in DMEM HG with sodium pyruvate (Biowest, Nauille, France) supplemented with 10% fetal bovine serum (FBS) (Thermo Fisher Scientific), 100 U/mL penicillin and 100 µg/mL streptomycin (Thermo Fisher Scientific). U2OS cell lines stably transfected with GFP-myc-tagged FGF2, GFP-myc-tagged FGF8, GFP-myc-tagged FGF12a, and myc-tagged FGF1 were prepared as previously described [11, 12] and cultured under the same conditions with the addition of 1.5 mg/mL geneticin (BioShop, G-418). U2OSFGF12a-SBP, U2OS-FGF12b-SBP, U2OS-FGF12b-GFP-myc, U2OS-FGF12aY126A-GFPmyc, U2OS-FG12aH1-GFP-myc, U2OS-FGF12aH2-GFP-myc and U2OS-FGF8N-GFP-myc cell lines were prepared as previously described [11, 12], using constructs in pcDNA3.1 vectors obtained from GeneUniversal (Newark, DE, USA). All cells were cultured in an atmosphere of 5% CO_2_ at 37 °C.

### FGF12 secretion analysis

0.2 million cells of individual U2OS-FGF-GFP-myc line were serum-starved or kept in complete media for 24 h and then incubated in fresh serum-free media or in complete media at 42 °C or at 37 °C for 2 h in the presence or absence of specific inhibitors: cyclosporin A (100 nM), ouabain octahydrate (10 µM) or LFM-A13 (275 µM). Media from above the cells were collected and centrifuged for 5 min at 1000 x g at 4 °C. Supernatants were incubated with Pierce anti-myc-tag magnetic beads for 2 h at 4 °C with rotation. The beads were then washed with PBS and the proteins were eluted with Laemmli sample buffer. Cell lysates and pull-down elutions were analyzed by western blotting with anti-GFP antibody. Results were quantified using Fiji Software [22].

### Exosome isolation

One million U2OS and U2OS-FGF12a-GFP-myc cells each were serum-starved for 24 h and then incubated in fresh serum-free medium at 42 °C for 2 h. The media from above the cells were collected and centrifuged several times at 4 °C discarding the pellet (5 min at 200 x g, 10 min at 1800 x g, 30 min at 3200 x g). The pellet was discarded, and the cleared media were transferred to the ultracentrifugation tube and centrifuged for 140 min at 62,000 x g at 4 °C using an Optima MAX-XP ultracentrifuge (Beckman-Coulter, Brea, CA, USA). The supernatant was discarded while the pellet was resuspended in PBS and centrifuged for 140 min at 62,000 x g at 4 °C. Pellet containing the exosomes was resuspended in PBS and incubated with Pierce anti-myc-tag magnetic beads for 2 h at 4 °C with rotation. Eluted proteins were analyzed by western blotting using anti-GFP and anti-HSP70 antibodies.

### Pull-down

To confirm the interaction between FGF12a, FGF12b, and ATP1A1, U2OS-FGF12a-SBP and U2OS-FGF12b-SBP cells were lysed in lysis buffer (150 mM NaCl, 40 mM Tris, 0.1% Triton, 2 mM EDTA, pH 7.5) supplemented with protease inhibitor cocktails (Roche) and incubated with Pierce streptavidin magnetic beads for 2 h at 4 °C. The resin was washed twice with lysis buffer, and bound proteins were eluted with Laemmli sample buffers. Proteins were analyzed by western blotting with anti-ATP1A1 and anti-SBP antibodies.

### Proximity ligation assay

The interaction between FGF12a, FGF12b, and ATP1A1 was assessed using DuoLink^®^ InSitu Fluorescence Protocol (Sigma-Aldrich). U2OS-FGF12a-GFP-myc and U2OS-FGF12b-GFP-myc cells were fixed with 4% paraformaldehyde (PFA) and permeabilized with 0.1% Triton X-100. Cells were then stained with antibodies against GFP and ATP1A1 in combination with secondary PLA probes and treated according to the manufacturer’s protocol. Cellswere stained with NucBlue Live ReadyProbes Reagent (#R37605) (Thermo Fischer Scientific) to visualize cell nuclei and with HSC CellMask Deep Red Stain (#H32721)(Thermo Fischer Scientific). Confocal fluorescence microscopy measurements were performed using an Opera Phenix High-Content Screening system (Perkin Elmer, Waltham, MA, USA). Cells were imaged with 63x Water NA 1.15 objective with binning 2 using two peaks autofocus and 2160 × 2160 px Camera ROI. There were 37 fields per well with XYZ z-stack per field at 0.5-µm intervals. Images were obtained and analyzed using Harmony high-content imaging and analysis software version 5.1 (Perkin Elmer).

### SiRNA transfection

0.1 million cells of each U2OS-FGF-GFP-myc cell line were transfected with 50 nM siRNA targeting ATP1A1 (#s1718), ATP1B1 (#s1734) or Tec kinase (#383) (Thermo Fisher Scientific) or non-targeting control siRNA (#D-001810-01-50) (Horizon Discovery, Cambridge, UK) using DharmaFECT transfection reagent (Horizon Discovery) according to the manufacturer’s instructions. After 24 h, the transfection medium was replaced with complete medium, and the cells were incubated for an additional 48 h, followed by secretion experiments. siRNA knock-down efficacy was evaluated using western blotting with anti-ATP1A1, anti-ATP1B1 and anti-Tec antibodies, and quantified with Fiji Software [22].

### Transient transfection

Transfection of U2OS cells with FGF12a-GFP-myc, FGF12aY126A-GFP-myc, FGF12aH1-GFP-myc, FGF12aH2-GFP-myc, FGF2-GFP-myc, FGF8-GFP-myc, FGF8N-GFP-myc pcDNA3.1 constructs was performed using FuGene HD transfection reagent (Promega, WI, USA) according to the manufacturer’s protocol. Experiments were performed 72 h after transfection. Transfection levels were analyzed by western blotting using anti-GFP antibody.

### Production and purification of recombinant proteins

Recombinant FGF2 and FGF12b were purified as previously described [11]. Recombinant His-tagged FGF12a was expressed in *E. coli* BL21 CodonPlus (DE3) RIL at 16 °C and purified from insoluble fraction using affinity chromatography with heparin resin (GE Healthcare) (Chicago, IL, USA).

### Lipid-protein interaction assay

Membrane Lipid Strips (#P-6002) (Echelon Biosciences, Salt Lake City, UT, USA) were incubated with His-tagged recombinant proteins according to the manufacturer’s protocol. Membrane Lipid Strips were analyzed using anti-His antibody.

### Droplet formation assay

5 µM of recombinant FGF12a, FGF12b and FGF2 were incubated with 10% PEG-8000 (#P2139) (Sigma-Aldrich) and heparin (100 nM) on ice for 30 min. Then, 2 µl droplets were loaded onto the glass coverslips and differential interference contrast (DIC) images were acquired using a Zeiss Axio Observer Z1 fluorescent microscope (Zeiss, Oberkochen, Germany) with an LD-Plan-Neofluar 40×/0.6 Korr M27 objective and Axiocam 503 camera. Images were analyzed using ZEN 2.3 software (Zeiss).

### Statistical analysis

All experiments were performed in at least three independent repeats. Statistical significance was determined with a two-tailed unpaired Student’s t-test using SigmaPlot software (Systat Software, Richmond, CA, USA). Statistical significance was set as follows: *p ≤ 0.05, **p ≤ 0.01, and ***p ≤ 0.001.

## Results

### FGF12a secretion is not dependent on ABC transporters nor does it occur through organelle-based translocation

**Fig. 1:**
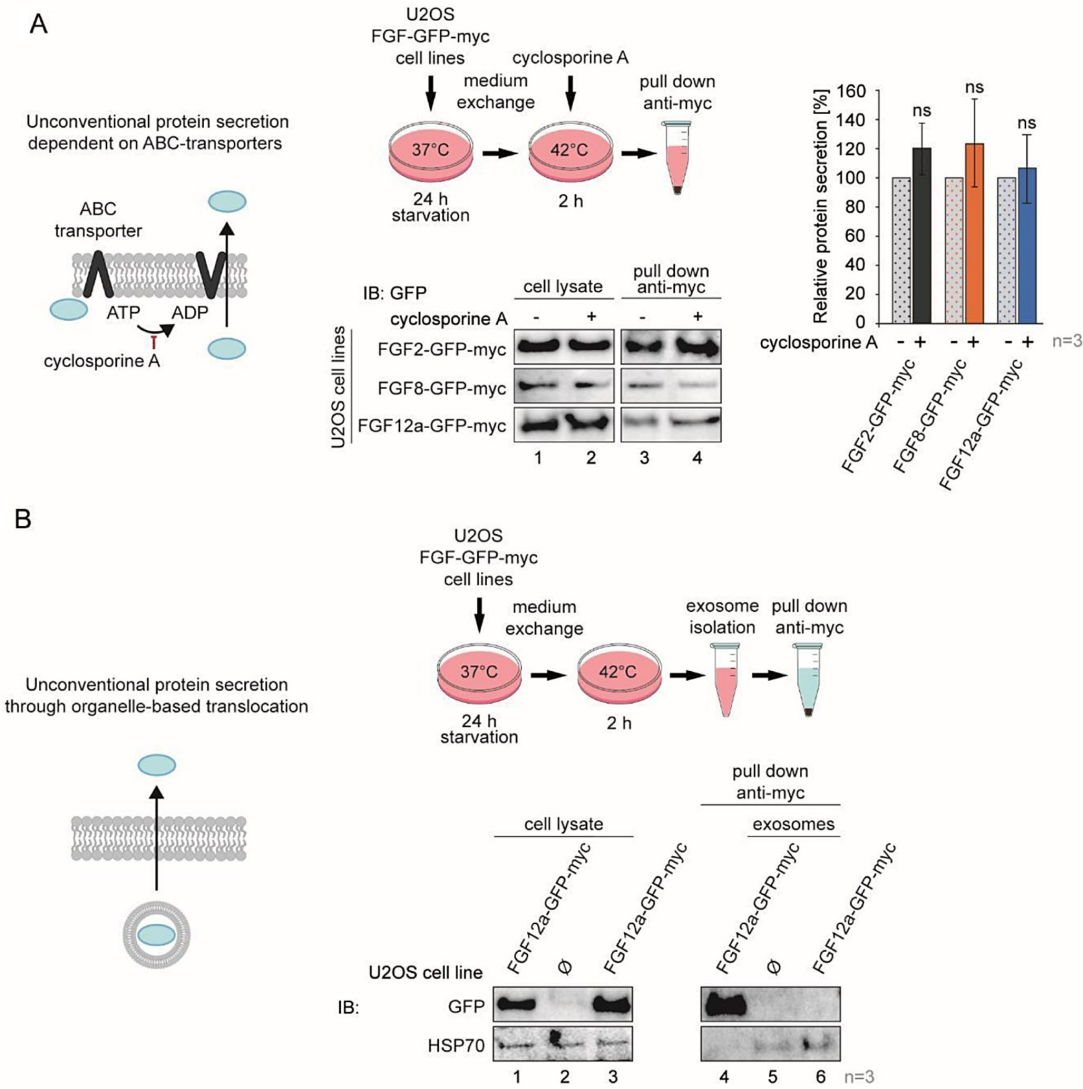
FGF12 is released from cells independently of ABC-transporters and organelle-based translocation. **a** U2OS-FGF-GFP-myc cells were serum-starved at 37 °C for 24 h. After the medium exchange, the cells were incubated at 42 °C for 2 h without or with the addition of cyclosporine A (100 nM) and the media from above the cells were collected, centrifuged and incubated with anti-myc tag magnetic beads. Elutions and lysates were analyzed by SDS-PAGE and western blotting using anti-GFP antibody. The graph shows relative protein secretion quantified using densitometric measurements in Fiji software. Student’s t-test was used for statistical analysis; ns *p* > 0.05. **b** U2OS and U2OS-FGF12a- GFP-myc cells were serum-starved at 37 °C for 24 h. After the medium exchange, cells were incubated at 42 °C for 2 h. The media from above the cells were collected and either incubated with anti-myc magnetic beads or differentially centrifuged to obtain the exosomal fraction. Exosomes were resuspended in PBS and condensed on anti-myc magnetic beads. Protein levels in media and exosomes were analyzed by SDS-PAGE and western blotting using anti-GFP antibody. The anti-HSP70 antibody was used as a control to confirm the successful isolation of exosomes

To investigate whether secretion of FGF12a is dependent on ABC-transporters (UPS II), we used U2OS cell lines stably transfected with FGF2, FGF8 and FGF12a in fusion with mGFP and myc-tag (U2OS-FGF2-GFP-myc, U2OS-FGF8-GFP-myc, U2OS-FGF12a-GFP-myc) and treated them with the UPS II inhibitor, cyclosporin A. FGF8, which has a classical signal peptide for secretion, and FGF2 secreted in an unconventional way served as controls. To achieve high levels of FGFs secretion cells were serum-starved for 24 h and then to induce secretion incubated in fresh serum-free medium for an additional 2 h at 42 °C [13, 14], with 100 nM of cyclosporin A. Proteins from the cell culture medium were captured on anti-myc magnetic beads and their amounts were analyzed by western blotting followed by densitometric measurements. We did not detect a statistically significant difference in the secretion levels of any of the studied proteins in the presence and absence of cyclosporin A (Fig. 1a).

We then tested whether FGF12a is secreted *via* organelle-based translocation by isolating exosomes from the medium from cells treated for 2 h at 42 ºC, using differential centrifugation. We compared FGF12a levels in exosomes and pull-down from the medium [23]. HSP70 was used as a control for proper exosome isolation [24]. The absence of FGF12a in purified exosomes indicates that its secretion is not based on organelle translocation (Fig. 1b).

### The A1 subunit of Na(+)/K(+) ATPase is responsible for the secretion of FGF12a

**Fig. 2:**
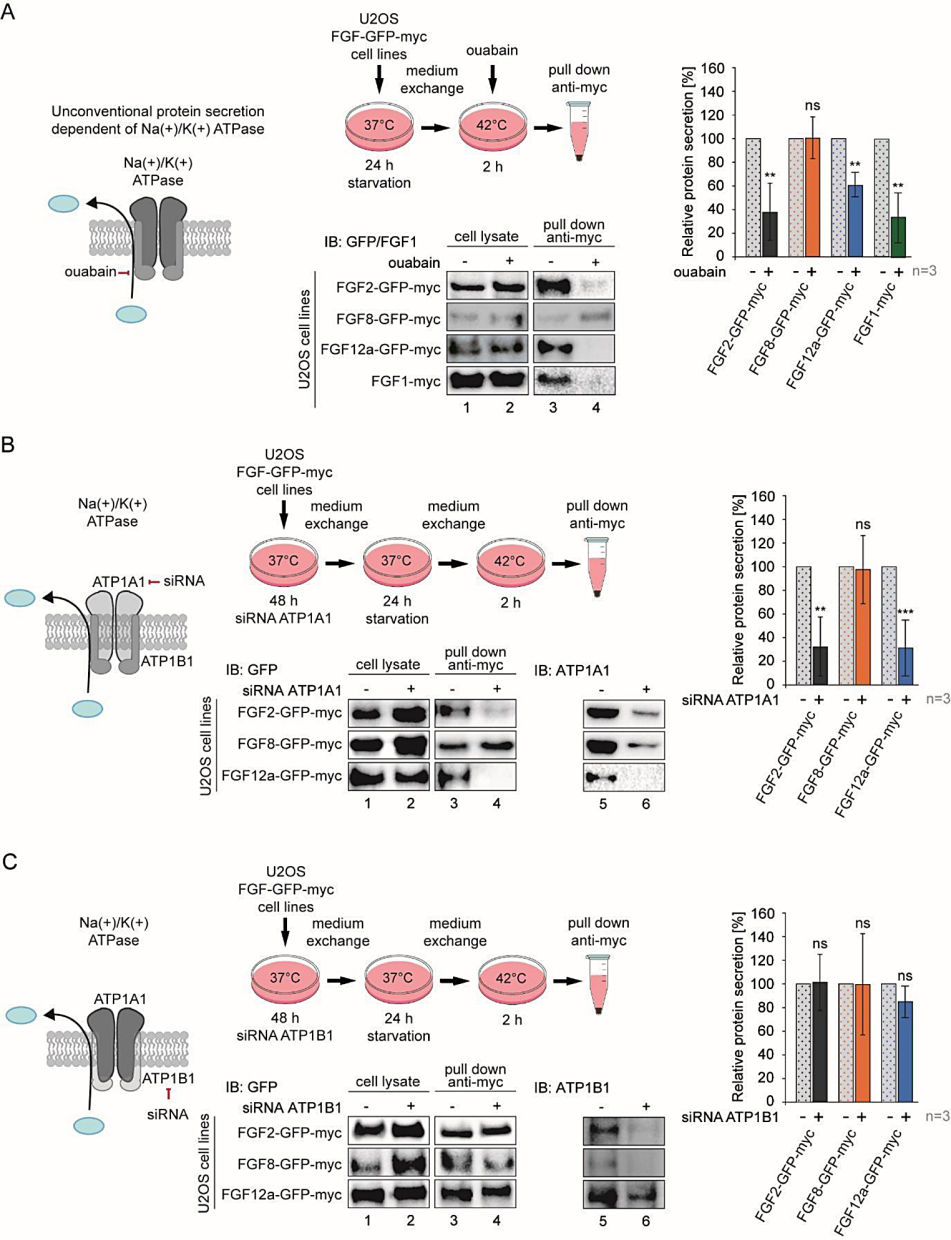
The interaction between FGF12a and ATP1A1 is crucial for FGF12a secretion. **a** U2OS-FGF2-GFP-myc, U2OS-FGF8-GFP-myc, U2OS-FGF12a-GFP-myc and U2OS-FGF1-myc cells were serum-starved at 37 °C for 24 h. After the medium exchange, the cells were incubated at 42 °C for 2 h without or with ouabain (10 µM) and the media from above the cells were collected, centrifuged, and incubated with anti-myc tag magnetic beads. Elutions and lysates were analyzed by SDS-PAGE and western blotting using anti-GFP antibody. The graph shows relative protein secretion quantified using densitometric measurements in Fiji software. Student’s t-test was applied for statistical analysis; ns *p* > 0.05, ** *p* ≤ 0.01. **b** and **c** U2OS-FGF2-GFP-myc, U2OS-FGF8-GFP-myc and U2OS-FGF12a-GFP-myc cells were transfected with siRNA targeting ATP1A1, ATP1B1 or with scramble siRNA (control) and serum-starved at 37 °C for 24 h after 48 h. The media were exchanged and the cells were incubated at 42 °C for 2 h. The media from above the cells were collected, centrifuged and incubated with anti-myc tag magnetic beads. Eluted proteins and lysates were analyzed by SDS-PAGE and western blotting using anti-GFP antibody. The anti-ATP1A1 and anti-ATP1B1 antibodies were used to verify knock-down effectiveness. Graphs show relative protein secretion quantified using densitometric measurements in Fiji software. Student’s t-test was applied for statistical analysis; ns *p* > 0.05, ** *p* ≤ 0.01, *** *p* ≤ 0.001

Using cell lines stably expressing the proteins: FGF1-myc, FGF2-GFP-myc, FGF8-GFP-myc, FGF12a-GFP-myc and Na(+)/K(+) ATPase inhibitor, ouabain, we verified the role of this ATPase in the secretion of individual FGF proteins. We showed that Na(+)/K(+) ATPase is crucial for the secretion of FGF2, FGF12a and, interestingly, also FGF1, while the level of secreted FGF8 remained the same regardless of the presence of the inhibitor (Fig. 2a).

Secretion experiments were performed under stress conditions (starvation and elevated temperature) because our previous studies have shown that they significantly increase the efficiency of FHF secretion [12], and because FGF1 is only secreted from cells under stress conditions [25, 26]. Also for FGF2, which does not require stress for secretion, adverse conditions increase the export of this protein from the cell [27]. To confirm the optimal choice of conditions for the secretion experiments, we performed control experiments analysing the secretion of FGF1, FGF2, FGF8 and FGF12 from cells that were not subjected to serum starvation and heat shock (Fig. S1). Consistent with our previous work, we observed no secretion of FGF1 from cells not subjected to stress. As expected, the levels of FGF2 and FGF12 in the media from unstressed cells were significantly lower than in starved cells at 42 °C. In contrast, the level of FGF8, a classically secreted FGF protein, in the medium was the same regardless of conditions (Fig. S1).

To verify which Na(+)/K(+) ATPase subunit is crucial for FGF12a secretion, we used siRNA specific for A1 and B1 subunits. We knocked-down each Na(+)/K(+) ATPase subunit in the following lines: U2OS-FGF2-GFP-myc, U2OS-FGF8-GFP-myc, U2OS-FGF12a-GFP-myc. Based on western blot analysis, we estimated that the A1 subunit (AT1PA1) was at least 80% silenced, and subunit B1 (ATP1B1) was silenced in 60% (Fig. 2b and c). Knock-down of the subunit A1 strongly inhibited the secretion of both FGF2 and FGF12a, while the level of FGF8 present in the cell medium remained unchanged (Fig. 2b). In contrast, silencing the B1 subunit did not affect the secretion level of either FGF protein (Fig. 2c). The data obtained indicate that only the A1 subunit of Na(+)/K(+) ATPase is critical for FGF12a secretion.

Our results are consistent with previous work by others showing that the A1 subunit is crucial for FGF2 secretion and that this interaction enables FGF2 to approach the inner plasma membrane [28].

### FGF12a interacts with the A1 subunit of Na(+)/K(+) ATPase

**Fig. 3:**
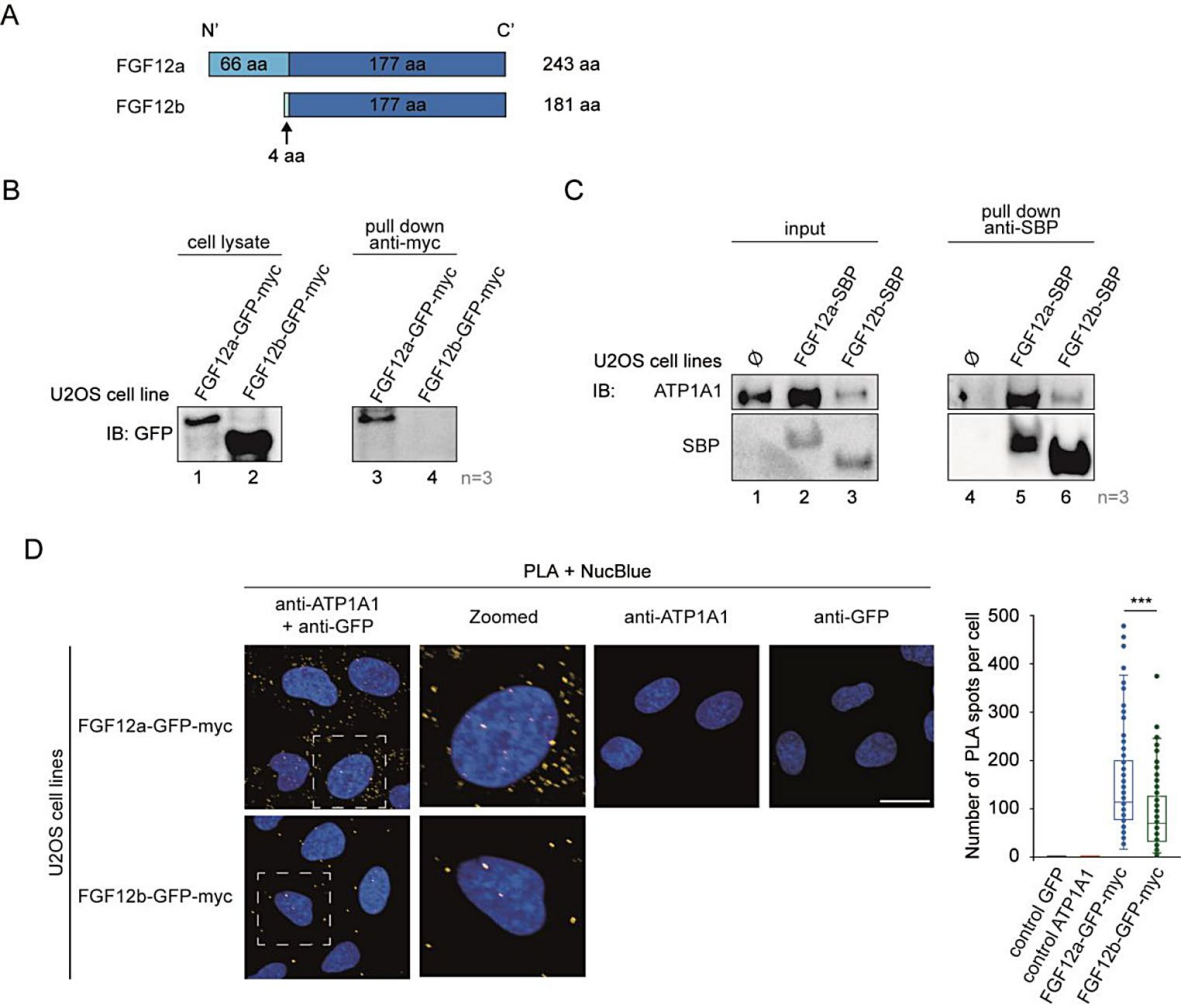
The short “b” isoform of FGF12 is not secreted due to weaker binding to ATP1A1. **a** Schematic illustration of FGF12 isoforms. **b** U2OS-FGF12a-GFP-myc and U2OS-FGF12b-GFP-myc cells were serum-starved at 37 °C for 24 h. After the medium exchange, cells were incubated at 42 °C for 2 h. The media from above the cells were collected, centrifuged and incubated with anti-myc tag magnetic beads. Elutions and lysates were analyzed by SDS-PAGE and western blotting using anti-GFP antibody. **c** U2OS-FGF12a-SBP and U2OS-FGF12b-SBP cell lysates were incubated with streptavidin magnetic beads for 2 h in 4 °C with rotation. The beads were washed twice with lysate buffer and the proteins were eluted with Laemmli buffer. The eluted proteins were analyzed by SDS-PAGE and western blotting using anti-SBP and anti-ATP1A1 antibodies. **d** Fluorescence images (maximum projection) of a representative PLA experiment using anti-GFP and anti-ATP1A1 antibodies in U2OS-FGF12a-GFP-myc and U2OS-FGF12b-GFP-myc cells. Cell nuclei were labeled with NucBlue Live. The PLA signal from the cytoplasm from 37 planes per well was quantified and divided by the number of cells in each plane. The images were analyzed using Harmony High-Content Imaging and Analysis Software. The scale bar represents 25 μm. Data shown in the graphs are PLA signals in cytoplasm per cell from representative experiments. Student’s t-test was applied for statistical analysis, * *p* ≤ 0.05, *** *p* ≤ 0.001. The experiment was repeated three times

FGF12 exists in two isoforms, “a” (long) and “b” (short), which differ in the N-terminal region (Fig. 3a, Fig. S2a) and show differential localization [1]. Using the stable U2OS-FGF12a-GFP-myc and U2OS-FGF12b-GFP-myc lines, we examined whether both FGF12 isoforms are secreted and found that only the long isoform (FGF12a) is present in the medium, while the short isoform (FGF12b) is not exported from the cell (Fig. 3b). We also verified that FGF12b is absent in the medium from cells that have not been subjected to cellular stress (Fig. S1).

We then examined whether this effect could be related to the interaction of specific FGF12 isoforms with ATP1A1. Using U2OS-FGF12a-SBP and U2OS-FGF12b-SBP cell lysates incubated with streptavidin magnetic beads, we showed that FGF12a efficiency of co-precipitation with ATP1A1, compared to FGF12b, is much stronger (Fig. 3c). We verified this result with a proximity ligation assay using anti-GFP and anti-ATP1 A1 antibodies and Opera Phenix High-Content Screening system microscope. The results were quantified with Harmony high-content imaging and analysis software. The data obtained confirms that FGF12a binds to ATP1A1 more effectively than FGF12b, as we observed 50% fewer PLA spots in cells with isoform “b” than in cells with isoform “a” (Fig. 3d). To verify that the PLA signal is located inside the cells, we additionally stained the cells with CellMask. Analysis of a sliced image of cells reveals that the interaction between ATP1A1 and FGF12 occurs in close proximity to cell membrane (Fig. S2b).

### Tec kinase is involved in the secretion of FGF12a

**Fig. 4:**
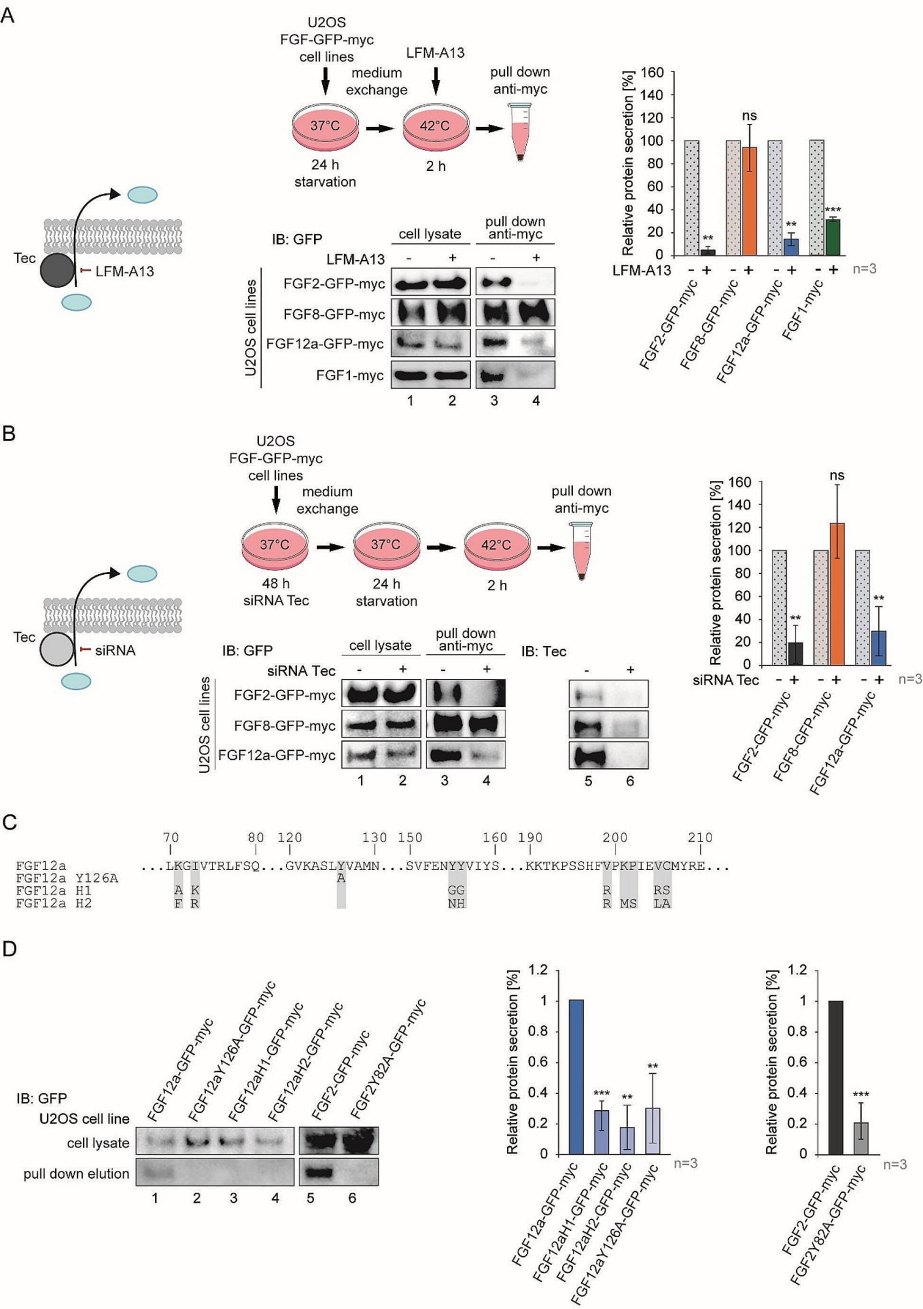
Tec kinase is involved in the secretion of FGF12a. **a** U2OS-FGF2-GFP-myc, U2OS-FGF8-GFP-myc, U2OS-FGF12a-GFP-myc and U2OS-FGF1-myc cells were serum-starved at 37 °C for 24 h. The media were exchanged, and the cells were incubated at 42 °C for 2 h. The media from above the cells were collected, centrifuged, and incubated with anti-myc tag magnetic beads. The eluted proteins and lysates were analyzed by SDS-PAGE and western blotting using anti-GFP antibody. The graph shows the relative secretion of proteins quantified using densitometric measurements in Fiji software. Student’s t-test was applied for statistical analysis; ns *p* > 0.05, ** *p* ≤ 0.01, *** *p* ≤ 0.001. **b** U2OS-FGF2-GFP-myc, U2OS-FGF8-GFP-myc and U2OS-FGF12a-GFP-myc cells were transfected with siRNA targeting Tec kinase or scramble siRNA (control) and, after 48 h, the cells were serum-starved at 37 °C for 24 h. After the media exchange, the cells were incubated at 42 °C for 2 h. The media from above the cells were collected, centrifuged and incubated with anti-myc tag magnetic beads. The eluted proteins and lysates were analyzed by SDS-PAGE and western blotting using anti-GFP antibody. The anti-Tec antibody was used to verify knock-down effectiveness. The graph shows the relative protein secretion quantified using densitometric measurements in Fiji software. Student’s t-test was applied for statistical analysis; ns *p* > 0.05, ** *p* ≤ 0.01. **c** Sequence alignment of wild type FGF12a and designed mutant variants. **d** U2OS cells were transiently transfected with plasmids encoding FGF12a-GFP-myc, FGF12aY126A-GFP-myc, FGF12aH1-GFP-myc, FGF12aH2-GFP-myc, FGF2-GFP-myc and FGF2Y82A-GFP-myc. 48 h after transfection, the media were exchanged and the cells were serum-starved at 37 °C for 24 h. The media were exchanged again and cells were incubated at 42 °C for 2 h. The media from above the cells were collected, centrifuged and incubated with anti-myc tag magnetic beads. The eluted proteins and lysates were analyzed by SDS-PAGE and western blotting using anti-GFP antibody. Graphs show the relative protein secretion quantified using densitometric measurements in Fiji software. Student’s t-test was applied for statistical analysis; **p ≤ 0.01, ***p ≤ 0.001

After interacting with the A1 subunit of Na(+)/K(+) ATPase, FGF2 interacts with Tec kinase, following which Tyr82 of FGF2 becomes phosphorylated, determining its efficiency of secretion [29]. Since Tec kinase is involved in the non-canonical secretion of FGF2, we tested whether this protein is also required for the export of FGF12a from the cell. Using cell lines stably expressing FGF2, FGF8, FGF12a, as well as FGF1 (U2OS-FGF2-GFPmyc, U2OS-FGF8-GFP-myc, U2OS-FGF12a-GFP-myc, and U2OS-FGF1-myc), we observed that inhibition of Tec kinase with LFM-A13 inhibitor caused a significant decrease in the levels of FGF1 (by 70%), FGF2 (by 96%) and FGF12a (by 88%) detected in the medium. At the same time, the amount of canonically secreted FGF8 remained the same regardless of the presence of the inhibitor (Fig. 4a). We verified these results using siRNA targeting Tec kinase, whose efficiency in silencing was about 80%. Similarly to the Tec inhibitor, Tec knock-down caused a significant decrease in the secretion levels of both FGF2 and FGF12a, while the amount of FGF8 remained unchanged (Fig. 4b).

### Identification of key sites for FGF12a secretion

Since our results suggest that Tec kinase is crucial in both the secretion of FGF2 and FGF12, we wanted to verify whether tyrosine residue 126, which corresponds to the Tyr82 residue crucial for FGF2 secretion [29], is also important for FGF12a secretion. To this end, we prepared FGF2 and FGF12a constructs in which the corresponding tyrosines were mutated to alanines (FGF2 Y82A and FGF12a Y126A) (Fig. 4c). In the next step, due to the fact that FGF2 is secreted in an oligomeric form, and there are reports of dimerization of FHF proteins, we also designed two FGF12a variants in which we mutated residues analogous to those responsible for dimerization of FGF13 (FGF12H1 and FGF12H2) (Fig. 4c) [7, 30]. Secretion of the FGF12a Y126A and H1 mutants was reduced by about 70%, and that of the H2 mutant by about 80% compared to the wild type (Fig. 4d). Such significantly reduced export efficiency of all three mutant variants suggests that the oligomeric state promotes a secretion process in which the Tyr126 residue additionally plays an important role.

### The N-terminal tail of FGF12 directs the protein to type II secretion

**Fig. 5:**
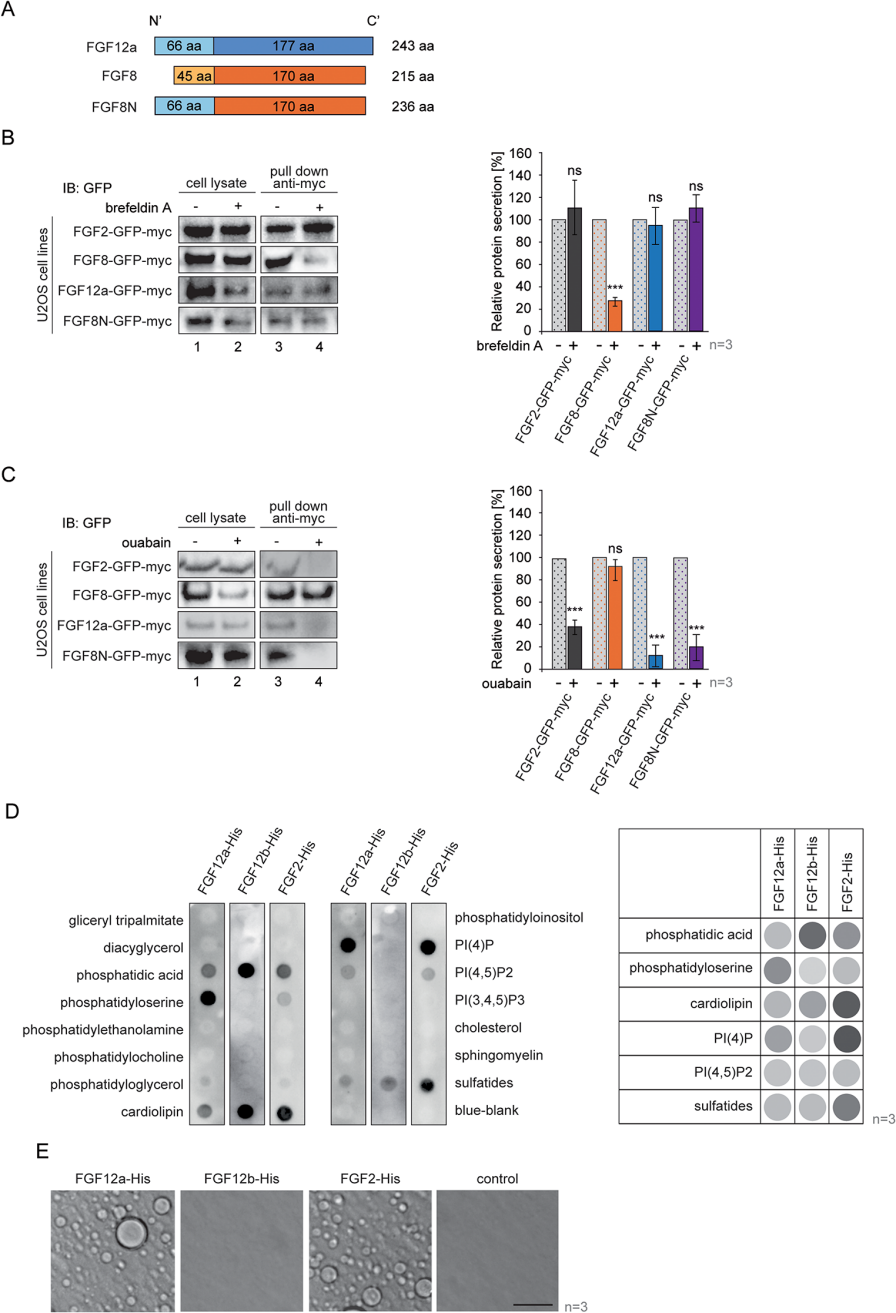
The N-terminal tail of FGF12a directs it to unconventional type II secretion, being crucial for its interaction with lipids and liquid-liquid phase separation. **a** Schematic illustration of an engineered mutant variant consisting of the core domain of FGF8 and the N-terminal tail of FGF12a – FGF8N. **b** and **c** U2OS cells were transiently transfected with plasmids encoding FGF2-GFP-myc, FGF8-GFP-myc, FGF12a-GFP-myc and FGF8N-GFP-myc. After 48 h, the media were exchanged and the cells were serum-starved at 37 °C for 24 h. The media were exchanged again and the cells were incubated at 42 °C for 2 h without or with brefeldin (2.5 µM) or ouabain (10 µM). The media from above the cells were collected, centrifuged and incubated with anti-myc tag magnetic beads. The eluted proteins and lysates were analyzed by SDS-PAGE and western blotting using anti-GFP antibody. Graphs show the relative protein secretion quantified using densitometric measurements in Fiji software. Student’s t-test was applied for statistical analysis; ns > 0.05 ***p ≤ 0.001. **d** Membrane lipid strips were incubated with FGF2-His, FGF12a-His and FGF12b-His. The membranes were then analyzed using an anti-His antibody. Representative results are shown on the left. The table on the right shows a schematic representation of the relative signal from bound protein to each lipid quantified using densitometric measurements in Fiji software. **e** DIC images of recombinant FGF12a-His, FGF12b-His and FGF2-His (5 µM) with 10% PEG 8000 and heparin (100 nM) after 30-min incubation on ice. Only FGF2 and FGF12a form liquid droplets, undergoing liquid-liquid phase separation

Since the short isoform of FGF12 is not secreted, this suggests that the N-terminal tail, which differentiates the long and short isoforms of this protein, may be crucial for unconventional type II secretion. To confirm this hypothesis, we designed a chimeric mutant consisting of the N-terminal tail of FGF12a and the core of FGF8 (without the signal peptide that directs the protein to the conventional secretion pathway), FGF8N (Fig. 5a). Using transiently transfected cells, we showed that the secretion of wild-type FGF8 is inhibited in the presence of brefeldin A, compound blocking the conventional ER/Golgi pathway of secretion, while the levels of both FGF2, FGF12a and FGF8N in media remained the same (Fig. 5b). When ouabain was added, blocking Na(+)/K(+) ATPase, only the level of wild-type FGF8 was unchanged, while the secretion of FGF2, FGF12a and the mutant variant FGF8N was significantly reduced by correspondingly: 63%, 89% and 81% (Fig. 5c). In addition, we showed that, similar for FGF2 and FGF12, the level of FGF8N secretion from unstressed cells is much lower than that from serum-starved cells incubated in 42 °C (Fig. S1). The results suggest that the N-terminal tail of FGF12a directs the protein to unconventional type II Na(+)/K(+) ATPase-dependent secretion.

### FGF12a interacts with lipids and undergoes LLPS

The final step in the unconventional secretion of FGF2 is its interaction with PI(4,5)P2, which triggers pore formation [30, 31]. We verified whether FGF12a, FGF12b and FGF2 interact with membrane lipids. FGF12a binds to phosphatidylserine, PI(4)P, PI(4,5)P2, phosphatidic acid, cardiolipin and sulfatides, while the short isoform FGF12b interacts only with phosphatidic acid, cardiolipin, and sulfatides. FGF2 interacts with phosphatidic acid, sulfatides, cardiolipin, PI(4)P, phosphatidylserine, and PI(4,5)P2 (Fig. 5d). Phosphatidylserine is a negatively charged phospholipid that is normally found in the inner leaflet of the plasma membrane [32]. However, it is exposed to the outer side of the membrane in many biological processes [33, 34] and has been shown to be involved in the unconventional secretion of FGF1 [35]. This suggests that it may be a carrier of FGF12a across the plasma membrane.

We then investigated whether FGF12a and FGF12b have liquid-liquid phase separation (LLPS) capabilities, similar to FGF2 [36]. LLPS is a process of molecular crowding, during which proteins, RNA and other biomolecules assemble into membrane-less compartments [37, 38]. The recombinant proteins were incubated with 10% PEG-8000 and heparin on ice for 30 min and then observed using DIC microscopy. For FGF12a and FGF2, we noticed spherical droplets in the solution, which were absent in the sample with FGF12b and in the protein-free control (Fig. 5e). This result suggests that the long isoform of FGF12 undergoes LLPS and that this process may be part of the pore formation inside the cell membrane, allowing FGF12 secretion from the cell.

## Discussion

Until recently, FHFs were thought to be exclusively intracellular and non-signaling proteins [1]. We showed for the first time that FHFs are in fact secreted, despite the absence of a signal peptide (SP), and act extracellularly through receptor binding and activation of downstream signaling [11, 12]. However, the molecular basis of their unconventional secretion was still unknown. In the present study, we have described in detail for the first time the secretion mechanism of FGF12a, the best-characterized representative of the FHF subfamily, identifying its major elements and interacting macromolecules, including proteins and lipids, required for this process.

SP-deficient proteins can leave cells through four different types of unconventional secretion – (I) direct translocation across the plasma membrane with pore formation, (II) secretion dependent on ABC-transporters activity, (III) secretion by intracellular vesicles or, (IV) in the case of transmembrane proteins, by Golgi bypass [13, 21]. Using the UPS type II inhibitor, cyclosporine A, which inhibits ABC-transporters [20], we demonstrated that FGF12a secretion is independent of cell membrane polarity resulting from ABC proteins activity. We then isolated exosomes from above the cells and verified that FGF12a is also not secreted by the type III UPS, which involves intracellular vesicles.

Our recent study suggests that FHFs may use a similar secretion mechanism as FGF2, which belongs to the canonical FGF group and also lacks SP. In both cases, secretion from the cell was inhibited in the presence of ouabain, an inhibitor of Na(+)/K(+) ATPase [12, 39]. Here, we showed that the addition of this inhibitor blocks the secretion of not only FGF2 and FGF12a, but also FGF1. As it has previously been shown that FGF1 secretion only occurs under stress conditions [25], in contrast to FGF2 secretion, which takes place in the absence of stress factors [13, 27], we decided to examine the secretion of the proteins studied both at 37 °C in complete medium and at 42 °C after starvation. We observed that secretion of all proteins tested, with the exception of the canonically secreted FGF8, was significantly less efficient at physiological temperature than at elevated temperature when cultured in serum-free medium (Fig. S1). Therefore, we conducted all further experiments under stress conditions. We then verified which Na(+)/K(+) ATPase subunit is crucial for FGF12a secretion by knock-down of each, A1 or B1, subunit of this protein. When the B1 subunit was silenced, the level of secreted FGFs did not change. However, with A1 subunit knock-down, the secretion of FGF2 and FGF12a decreased significantly.

In the next step, we tested whether both FGF12 isoforms are secreted from the cell. We found that the shorter “b” isoform, unlike FGF12a, remains inside the cell, regardless of a stress factor such as elevated temperature. To investigate the cause of this phenomenon, we examined whether both FGF12 isoforms could bind to ATP1A1. The co-precipitation experiment suggests that FGF12b bound significantly weaker than FGF12a to ATP1A1, which may be the reason why the “b” isoform is not secreted from cells.

Once recruited to the vicinity of the cell membrane by ATP1A1, FGF2 interacts with Tec kinase, which is an essential protein for its secretion [29]. Using the LFM-A13 inhibitor and a specific siRNA targeting Tec kinase, we showed that its presence and activity, are also critical for FGF12a secretion. In addition, we demonstrated for the first time that the presence of a Tec inhibitor also blocks the secretion of FGF1, for which a role for Tec kinase had not previously been suggested. In the case of FGF2, Tec kinase phosphorylates its Tyr82, providing a specific signal for its secretion [29]. The strongly reduced secretion of the FGF12a variant with an analogous mutation, Y126A, suggests the importance of this specific Tyr residue for the secretion of FGF family proteins *via* an unconventional pathway. Since FHFs have been shown to form dimers that appear to be crucial for their sodium channel regulating activity [7, 30], we decided to mutate several FGF12a residues potentially responsible for oligomerization. For the substitutions, we chose analogous positions to those indicated for FGF13 dimerization, resulting in FGF12aH1 and FGF12aH2 variants. Secretion of both variants was significantly reduced compared to wildtype FGF12a, suggesting that oligomerization of FGF12 is crucial for its efficient export from the cell, similar to that of FGF2 [30, 31].

In view of the lack of ability of the “b” isoform of FGF12 to be secreted from cells and the fact that the N-terminal tail of FHF proteins plays an important role in their binding to voltage-gated sodium channels, modulating resurgent currents [40], we hypothesized that a fragment at the N-terminus of FGF12a is critical for its unconventional secretion. We verified this assumption by engineering an FGF8 mutant, replacing its signal peptide for conventional secretion with an N-terminal fragment of FGF12a. This mutant was efficiently secreted from cells *via* a Na(+)/K(+) ATPase-dependent pathway, and its secretion was insensitive to the presence of brefeldin A, which inhibits the ER to Golgi-dependent pathway. Thus, we demonstrated that the N-terminal fragment of FGF12a is essential for its unconventional secretion.

Since the last step of secretion by direct translocation across the membrane (UPS I) is the formation of a pore inside the lipid bilayer, we checked with which lipids FGF12a, FGF12b and FGF2 proteins interact. Using membrane lipid strips, we observed that FGF12a and FGF2 bind to virtually the same lipids, while FGF12b does not bind to phosphatidylserine and phosphatidylinositol. Phosphatidylserine is a lipid that is normally found in the inner leaflet of the plasma membrane but can be “flipped” to the other side during certain processes, such as myogenesis, blood coagulation and apoptotic cell engulfment [33, 34]. The lack of interaction between FGF12b and this phospholipid may be the reason why FGF12b is not efficiently secreted from cells.

Looking for a mechanism for the direct passage of FGF12a across the membrane, we decided to verify its ability to form local condensations called liquid-liquid phase separations [38], a phenomenon recently described as important for FGF2 in signal transduction [36]. We have shown for the first time that FGF12a, like FGF2, has this ability, and we suggest that this process may be the final step in the formation of pores inside the plasma membrane that facilitate the exit of FGF proteins from the cell by unconventional secretion.

In summary, the long isoform of FGF12 is secreted from cells in an unconventional manner. We have identified the following components that are crucial for this process: the A1 subunit of Na(+)/K(+) ATPase, Tec kinase, phosphatidylserine and phosphatidylinositol. We also indicated which regions are required for FGF12 secretion and proposed that liquid-liquid phase separation may be the final step in the formation of pores inside the lipid bilayer. Based on the results, we suggest that all unconventionally secreted FGFs, lacking a signal peptide, utilize the same mechanism. In summary, the long isoform of FGF12 is secreted from cells in an unconventional manner.

### Electronic supplementary material

Below is the link to the electronic supplementary material.

Supplementary Material 1

## Data Availability

All data available within the article.

## References

[1] Olsen SK, Garbi M, Zampieri N, Eliseenkova AV, Ornitz DM, Goldfarb M, Mohammadi M (2003) Fibroblast growth factor (FGF) homologous factors share structural but not functional homology with FGFs. J Biol Chem 278:34226–34236. 10.1074/jbc.M303183200 12815063 10.1074/jbc.M303183200

[2] Pablo JL, Pitt GS (2016) Fibroblast growth factor homologous factors: New roles in neuronal health and disease. Neuroscientist 22:19–25. 10.1177/1073858414562217 25492945 10.1177/1073858414562217 PMC4555190

[3] Hanada Y, Nakamura Y, Ozono Y, Ishida Y, Takimoto Y, Taniguchi M, Ohata K, Koyama Y, Imai T, Morihana T, Konodo M, Sato T, Inohara H, Shimada S (2018) Fibroblast growth factor 12 is expressed in spiral and vestibular ganglia and necessary for auditory and equilibrium function. Sci Rep 8:11491. 10.1038/s41598-018-28618-0 30065296 10.1038/s41598-018-28618-0 PMC6068167

[4] Hartung H, Feldman B, Lovec H, Coulier F, Birnbaum D, Goldfarb M (1997) Murine FGF-12 and FGF-13: expression in embryonic nervous system, connective tissue and heart. Mech Dev 64:31–39. 10.1016/S0925-4773(97)00042-7 9232594 10.1016/S0925-4773(97)00042-7

[5] Munoz-Sanjuan I, Smallwood PM, Nathans J (2000) Isoform diversity among fibroblast growth factor homologous factors is generated by alternative promoter usage and differential splicing. J Biol Chem 275:2589–2597. 10.1074/jbc.275.4.2589 10644718 10.1074/jbc.275.4.2589

[6] Schoorlemmer J, Goldfarb M (2001) Fibroblast growth factor homologous factors are intracellular signaling proteins. Curr Biol 11:793–797. 10.1016/S0960-9822(01)00232-9 11378392 10.1016/S0960-9822(01)00232-9 PMC3216481

[7] Goetz R, Dover K, Laezza F, Shtraizent N, Huang X, Tchetchik D, Eliseenkova AV, Xu CF, Neubert TA, Ornitz DM, Goldfarb M, Mohammadi M (2009) Crystal structure of a fibroblast growth factor homologous factor (FHF) defines a conserved surface on FHFs for binding and modulation of voltage-gated sodium channels. J Biol Chem 284:17883–17896. 10.1074/jbc.M109.001842 19406745 10.1074/jbc.M109.001842 PMC2719427

[8] König HG, Fenner BJ, Byrne JC, Schwamborn RF, Bernas T, Jefferies CA, Prehn JHM (2012) Fibroblast growth factor homologous factor 1 interacts with NEMO to regulate NF-κB signaling in neurons. J Cell Sci 125:6058–6070. 10.1242/jcs.111880 23097049 10.1242/jcs.111880

[9] Schoorlemmer J, Goldfarb M (2002) Fibroblast growth factor homologous factors and the islet brain-2 scaffold protein regulate activation of a stress-activated protein kinase. J Biol Chem 277:49111–49119. 10.1074/jbc.M205520200 12244047 10.1074/jbc.M205520200 PMC4266389

[10] Sochacka M, Karelus R, Opalinski L, Krowarsch D, Biadun M, Otlewski J, Zakrzewska M (2022) FGF12 is a novel component of the nucleolar NOLC1/TCOF1 ribosome biogenesis complex. Cell Communication Signal 20:182. 10.1186/s12964-022-01000-4 10.1186/s12964-022-01000-4 PMC9677703 36411431

[11] Sochacka M, Opalinski L, Szymczyk J, Zimoch MB, Czyrek A, Krowarsch D, Otlewski J, Zakrzewska M (2020) FHF1 is a bona fide fibroblast growth factor that activates cellular signaling in FGFR-dependent manner. Cell Communication Signal 18:69. 10.1186/s12964-020-00573-2 10.1186/s12964-020-00573-2 PMC7193404 32357892

[12] Biadun M, Sochacka M, Karelus R, Baran K, Czyrek A, Otlewski J, Krowarsch D, Opalinski L, Zakrzewska M (2023) FGF homologous factors are secreted from cells to induce FGFR-mediated anti-apoptotic response. FASEB J 37:e23043. 10.1096/fj.202300324R 37342898 10.1096/fj.202300324R

[13] Nickel W, Rabouille C (2009) Mechanisms of regulated unconventional protein secretion. Nat Rev Mol Cell Biol 10:148–155. 10.1038/nrm2617 19122676 10.1038/nrm2617

[14] Pallotta MT, Nickel W (2020) FGF2 and IL-1β - explorers of unconventional secretory pathways at a glance. J Cell Sci 133:jcs250449. 10.1242/jcs.250449 33154173 10.1242/jcs.250449

[15] Rayne F, Debaisieux S, Bonhoure A, Beaumelle B (2010) HIV-1 Tat is unconventionally secreted through the plasma membrane. Cell Biol Int 34:409–413. 10.1042/cbi20090376 19995346 10.1042/cbi20090376

[16] Schatz M, Tong PBV, Beaumelle B (2018) Unconventional secretion of viral proteins. Semin Cell Dev Biol 83:8–11. 10.1016/j/semcdb.2018.03.008 29571970 10.1016/j/semcdb.2018.03.008

[17] Katsinelos T, Zeitler M, Dimou E, Karakatsani A, Muller HM, Nachhman E, Steringer JP, Ruiz de Almodovar C, Nickel W, Jakn TR (2018) Unconventional secretion mediates the trans-cellular spreading of tau. Cell Rep 23:2039–2055. 10.1016/j.celrep.2018.04.056 29768203 10.1016/j.celrep.2018.04.056

[18] Merezhko M, Uronen RL, Huttunen HJ (2020) The Cell Biology of Tau Secretion. Front Mol Neurosci 13:569818. 10.3389/fnmol.2020.569818 33071756 10.3389/fnmol.2020.569818 PMC7539664

[19] Sparn C, Dimou E, Meyer A, Saleppico R, Wegehingel S, Gerstner M, Klaus S, Ewers H, Nickel W (2022) Glypican-1 drives unconventional secretion of Fibroblast Growth factor 2. Elife 11:75545. 10.7554/eLife.75545 10.7554/eLife.75545 PMC8986318 35348113

[20] De Maio A, Hightower LE (1989) Heat shock proteins and the biogenesis of cellular membranes. Cell Stress Chaperones 26:769–783. 10.1007/s12192-020-01173-2/Published 10.1007/s12192-020-01173-2/Published PMC7736428 33083932

[21] Dimou E, Nickel W (2018) Current Biology unconventional mechanisms of eukaryotic protein secretion. Curr Biol 28:R406–410. 10.1016/j.cub.2017.11.074 29689224 10.1016/j.cub.2017.11.074

[22] Schindelin J, Arganda-Carreras I, Frise E, Kaynig V, Longair M, Pietzsch T, Preibisch S, Rueden C, Saalfeld S, Schmid B, Tinevez JY, White DJ, Hartenstein V, Eliceiri K, Tomancak P, Cardona A (2012) Fiji: an open-source platform for biological-image analysis. Nat Methods 9:676–682. 10.1038/nmeth.2019 22743772 10.1038/nmeth.2019 PMC3855844

[23] Chhoy P, Brown CW, Amante JJ, Mercurio AM (2021) Protocol for the separation of extracellular vesicles by ultracentrifugation from in vitro cell culture models. STAR Protoc 2:100303. 10.1016/j.xpro.2021.100303 33554138 10.1016/j.xpro.2021.100303 PMC7848770

[24] Komarova EY, Suezov RV, Nikotina AD, Aksenov ND, Garaeva LA, Shtam TA, Zhakhov AV, Martynova MG, Bystrova OA, Istomina MS, Ischenko AM, Margulis BA, Guzhova IV (2021) Hsp70-containing extracellular vesicles are capable of activating of adaptive immunity in models of mouse melanoma and colon carcinoma. Sci Rep 11:21314. 10.1038/s41598-021-00734-4 34716378 10.1038/s41598-021-00734-4 PMC8556270

[25] Jackson A, Friedman S, Zhan X, Engleka KA, Forough R, Maciag T (1999) Heat shock induces the release of fibroblast growth factor 1 from NIH 3T3 cells. Proc Natl Acad Sci 89:10691–10695. 10.1073/pnas.89.22.10691 10.1073/pnas.89.22.10691 PMC50407 1279690

[26] Mouta Carreira C, Landriscina M, Bellum S, Prudovsky I, Maciag T (2001) The comparative release of FGF1 by hypoxia and temperature stress. Growth Factors 18:277–285. 10.3109/08977190109029116 11519826 10.3109/08977190109029116

[27] Keller M, Rüegg A, Werner S, Beer HD (2008) Active Caspase-1 is a Regulator of unconventional protein secretion. Cell 132:818–831. 10.1016/j.cell.2007.12.040 18329368 10.1016/j.cell.2007.12.040

[28] Legrand C, Saleppico R, Sticht J, Lolicato F, Muller HM, Wegehingel S, Dimou E, Steringer JP, Ewers H, Vattulainen I, Freund C, Nickel W (2020) The Na,K-ATPase acts upstream of phosphoinositide PI(4,5)P2 facilitating unconventional secretion of Fibroblast Growth factor 2. Commun Biol 3:141. 10.1038/s42003-020-0871-y 32214225 10.1038/s42003-020-0871-y PMC7096399

[29] Ebert AD, Laußmann M, Wegehingel S, Kaderali L, Erfle H, Reichert J, Lechner J, Beer HD, Pepperkok R, Nickel W (2010) Tec-kinase-mediated phosphorylation of Fibroblast Growth factor 2 is essential for unconventional secretion. Traffic 11:813–826. 10.1111/j.1600-0854.2010.01059.x 20230531 10.1111/j.1600-0854.2010.01059.x

[30] Muller HM, Steringer JP, Wegehingel S, Bleicken S, Munster M, Dimou E, Unger S, Weidmann G, Andreas H, Garcoa-Saez AJ, Wild K, Sinning I, Nickel W (2015) Formation of disulfide bridges drives oligomerization, membrane pore formation, and translocation of fibroblast growth factor 2 to cell surfaces. J Biol Chem 290:8925–8937. 10.1074/jbc.M114.622456 25694424 10.1074/jbc.M114.622456 PMC4423683

[31] Steringer JP, Bleicken S, Andreas H, Zacherl S, Laussmann M, Temmerman K, Contreras FX, Bhart TAM, Lechner J, Muller HM, Briggs JAG, Garcia-Saex AJ, Nickel W (2012) Phosphatidylinositol 4,5-bisphosphate (PI(4,5)P 2)-dependent oligomerization of fibroblast growth factor 2 (FGF2) triggers the formation of a lipidic membrane pore implicated in unconventional secretion. J Biol Chem 287:27659–27669. 10.1074/jbc.M112.381939 22730382 10.1074/jbc.M112.381939 PMC3431657

[32] Murate M, Abe M, Kasahara K, Iwabuchi J, Umeda M, Kobayashi T (2015) Transbilayer distribution of lipids at nano scale. J Cell Sci 128:1627–1638. 10.1242/jcs.163105 25673880 10.1242/jcs.163105

[33] Hankins HM, Baldridge RD, Xu P, Graham TR (2015) Role of Flippases, scramblases and transfer proteins in phosphatidylserine subcellular distribution. Traffic 16:35–47. 10.1111/tra.12233 25284293 10.1111/tra.12233 PMC4275391

[34] Tsuchiya M, Hara Y, Okuda M, Itoh M, Nishioka R, Shiomi A, Nagao K, Mori M, Mori Y, Ikenouchi J, Suzuki R, Tanaka M, Ohwada T, Aoki J, Kanagawa M, Toda T, Nagata Y, Matsuda R, Takayama Y, Umeda M (2018) Cell surface flip-flop of phosphatidylserine is critical for PIEZO1-mediated myotube formation. Nat Commun 9:2049. 10.1038/s41467-018-04436-w 29799007 10.1038/s41467-018-04436-w PMC5967302

[35] Kirov A, Al-Hashimi H, Solomon P, Mazur C, Thorpe PE, Sims PJ, Tarantini F, Kumar TKS, Prudovsky I (2012) Phosphatidylserine externalization and membrane blebbing are involved in the nonclassical export of FGF1. J Cell Biochem 113:956–966. 10.1002/jcb.23425 22034063 10.1002/jcb.23425 PMC3264788

[36] Xue S, Zhou F, Zhao T, Zhao H, Wang X, Chen L, Li J, Luo SZ (2022) Phase separation on cell surface facilitates bFGF signal transduction with heparan sulphate. Nat Commun 13:1112. 10.1038/s41467-022-28765-z 35236856 10.1038/s41467-022-28765-z PMC8891335

[37] Uversky VN (2017) Intrinsically disordered proteins in overcrowded milieu: membrane-less organelles, phase separation, and intrinsic disorder. Curr Opin Struct Biol 44:18–30. 10.1016/j.sbi.2016.10.015 27838525 10.1016/j.sbi.2016.10.015

[38] Boeynaems S, Alberti S, Fawzi NL, Mittag T, Polymenidou M, Rousseau F, Schymkowitz J, Shorter J, Van Den Wolozin B, Tompa P, Fuxreiter M (2018) Protein phase separation: a New Phase in Cell Biology. Trends Cell Biol 28:420–435. 10.1015/j.tcb.2018.02.004 29602697 10.1015/j.tcb.2018.02.004 PMC6034118

[39] Emanuel JR, Schulzj J, Zhouj XM, Kent RB, Housman D, Cantley L, Levenson R (1988) Expression of an Ouabain-resistant Na,K-ATPase in CV-1 cells after transfection with a cDNA encoding the rat Na,K-ATPase a1 subunit. J Biol Chem 263:7726–7733 PMID:2836394 2836394 10.1016/S0021-9258(18)68559-X

[40] Xiao Y, Theile JW, Zybura A, Pan Y, Lin Z, Cummins TR (2022) A-type FHFs mediate resurgent currents through TTX-resistant voltage-gated sodium channels. Elife 11:e77558. 10.7554/eLife.77558 35441593 10.7554/eLife.77558 PMC9071269

