# Supplementary material for: Uncovering key steps in FGF12 cellular release reveals a common mechanism for unconventional FGF protein secretion
Source: Cell Mol Life Sci. 2024 Aug 19;81(1):356. doi: 10.1007/s00018-024-05396-9 (PMC11335280; doi:10.1007/s00018-024-05396-9)
Supplement: Supplementary file 1 — Supplementary Material 1 [file 18_2024_5396_MOESM1_ESM.docx]

Supplementary material

**Uncovering key steps in FGF12 cellular release reveals a common mechanism for unconventional FGF protein secretion**

Martyna Biadun^1^, Martyna Sochacka^2^, Marta Kalka^1^, Aleksandra Chorazewska^1^, Radoslaw Karelus^1^, Daniel Krowarsch^2^, Lukasz Opalinski^1,^ Malgorzata Zakrzewska^1^*

^1^Department of Protein Engineering, Faculty of Biotechnology, University of Wroclaw, Wroclaw, Poland

^2^Department of Protein Biotechnology, Faculty of Biotechnology, University of Wroclaw, Wroclaw, Poland

* Corresponding author, e-mail: malgorzata.zakrzewska@uwr.edu.pl

**Supplementary figure legends**

**Fig. S1.** **FGF protein secretion under physiological and stress conditions**. U2OS-FGF1-myc, U2OS-FGF2-GFP-myc, U2OS-FGF8-GFP-myc, U2OS-FGF12a-GFP-myc, U2OS-FGF12b-GFP-myc and U2OS-FGF8N-GFP-myc cells were incubated in media with or without serum at 37 °C for 24 h. After the medium exchange, cells were incubated in fresh media with or without serum at 37 °C or at 42 °C for 2 h. The media from above the cells were collected, centrifuged and incubated with anti-myc magnetic beads. Elutions and lysates were analyzed by SDS-PAGE and western blotting using anti-GFP antibody.

# Fig. S2. Analysis of the interaction of FGF12 isoforms with ATP1A1 protein. a Sequence alignment of FGF12a and FGF12b. * (asterisk) indicates fully conserved positions. b PLA analysis of FGF12 isoform interaction with ATP1A1. Fluorescence images (maximum projection and slice z = -0.5 μm) of a representative PLA experiment using anti-GFP and anti-ATP1A1 antibodies in U2OS-FGF12a-GFP-myc and U2OS-FGF12b-GFP-myc cells. Cells were stained with NucBlue Live to visualize cell nuclei and HSC CellMask Deep Red.

**
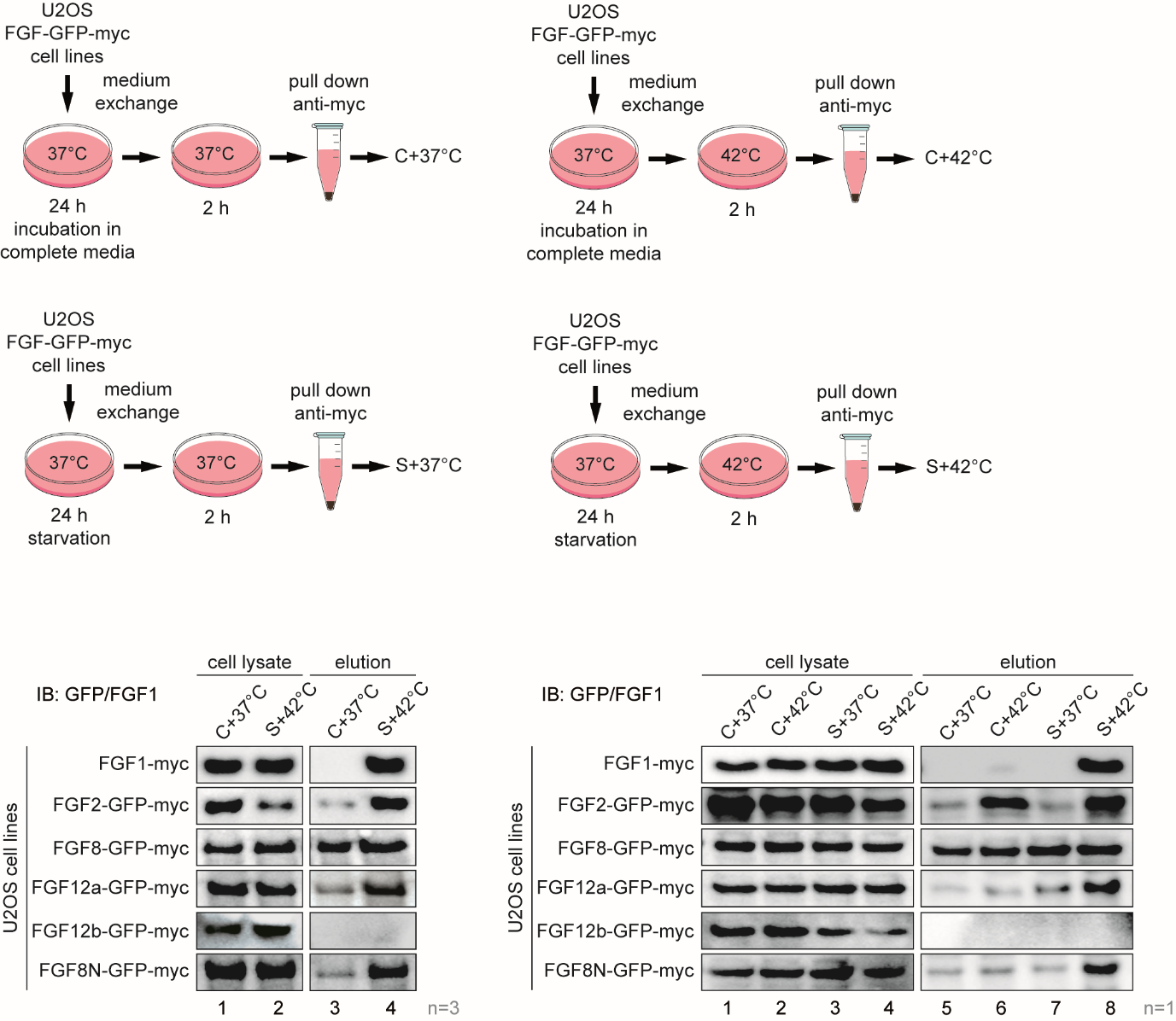
**

**Fig. S1**

**
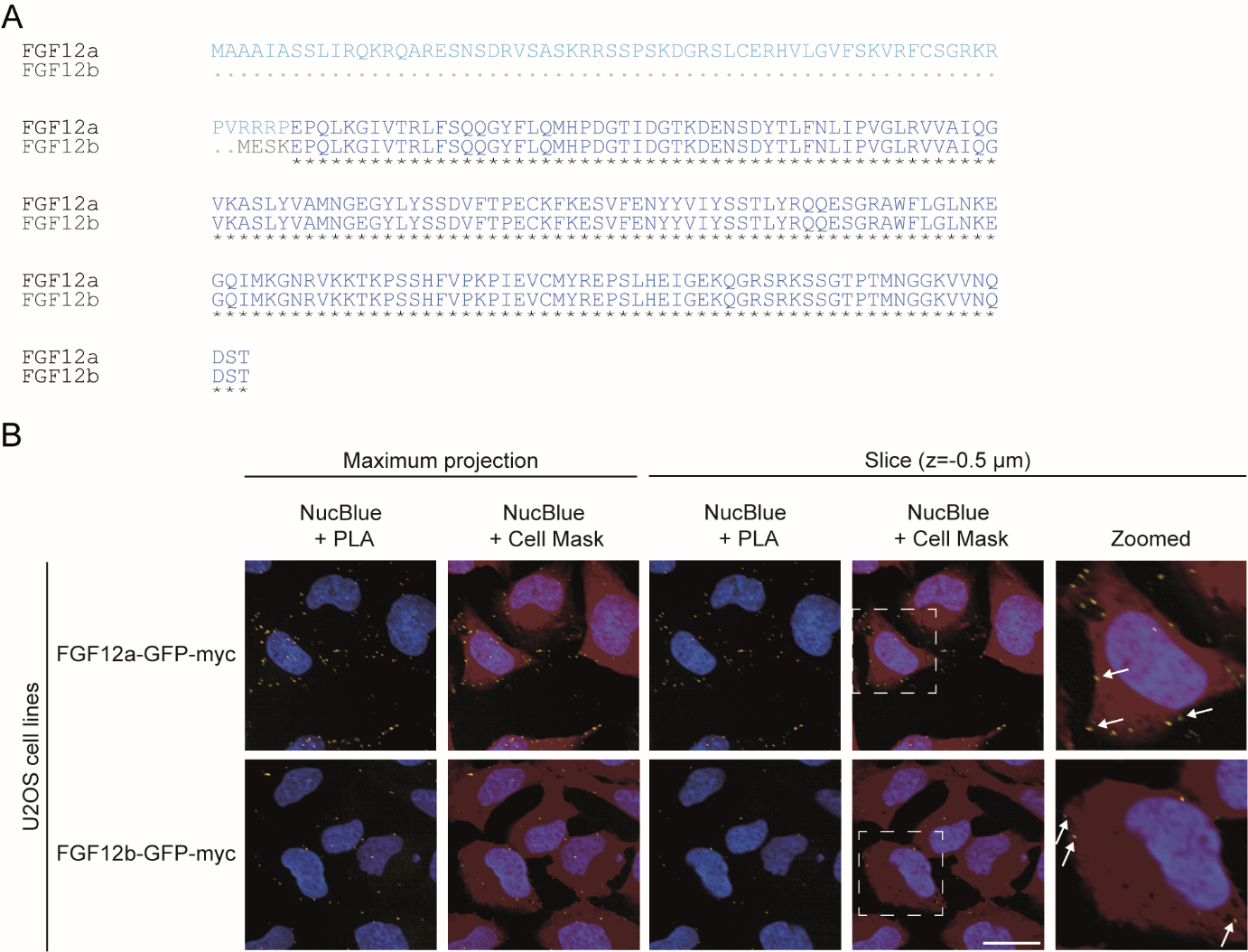
**

**Fig. S2**
